# Biological, pathological, and multifaceted therapeutic functions of exosomes to target cancer

**DOI:** 10.32604/or.2023.030401

**Published:** 2023-11-15

**Authors:** VIGNESH BALAJI E, DIVYA RAMESH, MANISHA CHUNGAN SHAJU, AKSHARA KUMAR, SAMYAK PANDEY, RAKSHA NAYAK, V. ALKA, SRISHTI MUNJAL, AMIR SALIMI, K. SREEDHARA RANGANATH PAI, SHANKAR M. BAKKANNAVAR

**Affiliations:** 1 Department of Pharmacology, Manipal College of Pharmaceutical Sciences, Manipal Academy of Higher Education, Manipal, Karnataka, 576104, India; 2 Department of Forensic Medicine and Toxicology, Kasturba Medical College, Manipal Academy of Higher Education, Manipal, Karnataka, 576104, India; 3 School of Health and Community Services, Durham College, Oshawa, Ontario, L1G2G5, Canada; 4 Department of Pharmaceutical Regulatory Affairs and Management, Manipal College of Pharmaceutical Sciences, Manipal Academy of Higher Education, Manipal, Karnataka, 576104, India; 5 Department of Clinical Psychology, Manipal College of Health Professions, Manipal Academy of Higher Education, Manipal, Karnataka, 576104, India; 6 Department of Speech and Hearing, Manipal College of Health Professions, Manipal Academy of Higher Education, Manipal, Karnataka, 576104, India; 7 Department of Pharmacy Practice, Manipal College of Pharmaceutical Sciences, Manipal Academy of Higher Education, Manipal, Karnataka, 576104, India

**Keywords:** Exosomes, Physiology, Cancer, Therapeutics, Challenges

## Abstract

Exosomes, small tiny vesicle contains a large number of intracellular particles that employ to cause various diseases and prevent several pathological events as well in the human body. It is considered a “double-edged sword”, and depending on its biological source, the action of exosomes varies under physiological conditions. Also, the isolation and characterization of the exosomes should be performed accurately and the methodology also will vary depending on the exosome source. Moreover, the uptake of exosomes from the recipients’ cells is a vital and initial step for all the physiological actions. There are different mechanisms present in the exosomes’ cellular uptake to deliver their cargo to acceptor cells. Once the exosomal uptake takes place, it releases the intracellular particles that leads to activate the physiological response. Even though exosomes have lavish functions, there are some challenges associated with every step of their preparation to bring potential therapeutic efficacy. So, overcoming the pitfalls would give a desired quantity of exosomes with high purity.

## Introduction

**FIGURE 1:**
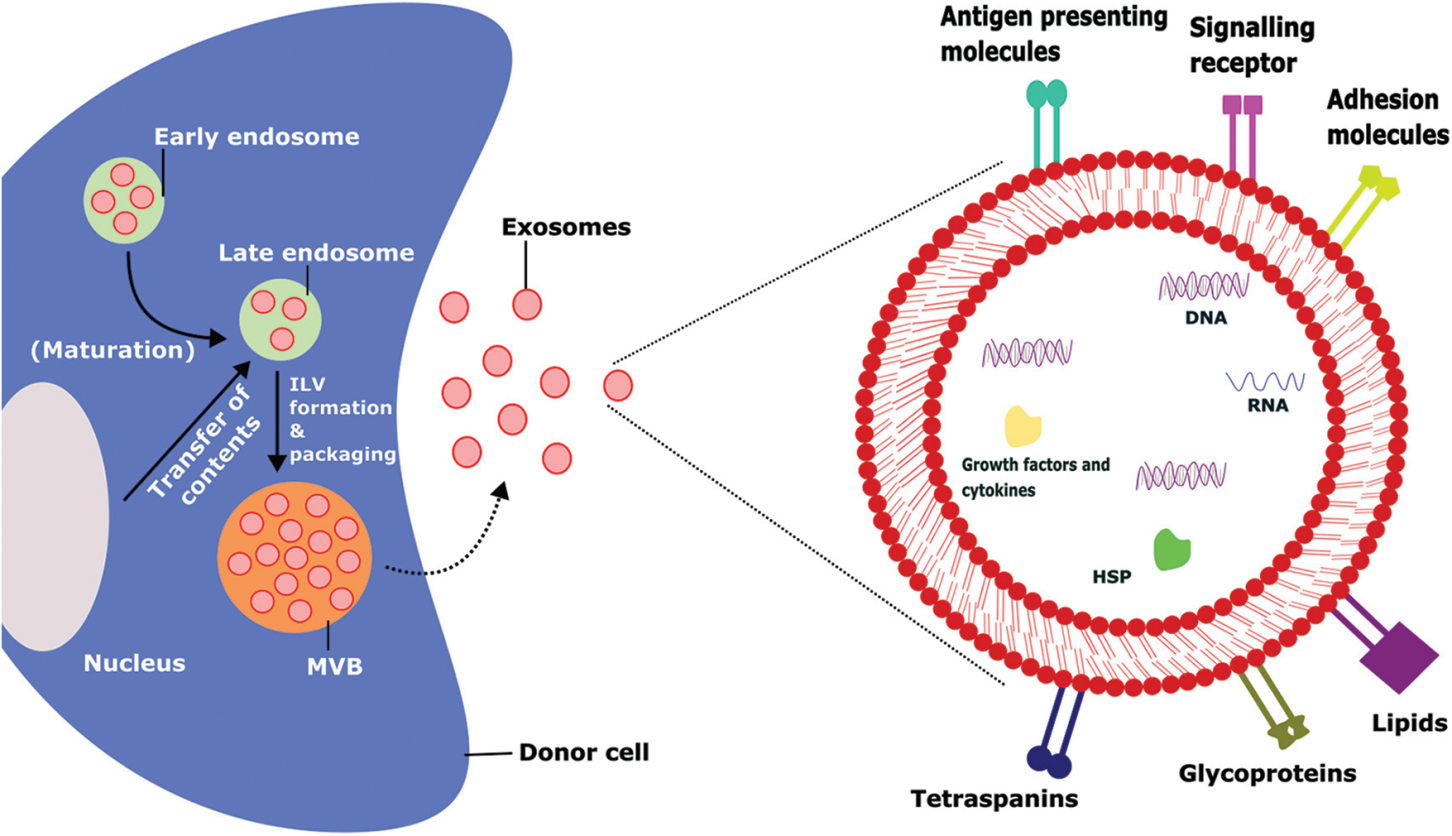
Biogenesis, intracellular contents, and surface markers of exosomes.

Extracellular vesicles (EVs) are tiny membrane vesicles that cells actively discharge to the systemic circulation. Exosomes and microvesicles are examples of similar-sized vesicles that can be further categorized based on their biogenesis, size, and biochemical features [1]. Exosomes are small membrane microvesicles generated from endosomes (Fig. 1) that have gained a lot of interest in the last decade due to their ability to transport cargo and act as therapeutic agents for various diseases [2]. Earlier, it was assumed that exosomes were regarded as the cellular debris released from the cells due to cellular damage. These are believed to have no significant effect on the neighboring cells. Recently, it was found that exosomes carry cargos that include proteins, lipids, nucleic acids, etc., [2,3]. Exosomes are considered a novel intercellular communication agent that could be important in various cellular activities [4,5]. Due to their endosomal origin, exosomes are abundant in endosomal membrane markers such as CD63, CD9, and CD81 [6]. Exosomes could be isolated from various laboratory techniques such as ultracentrifugation, size-based filtration, immune-affinity purification techniques, and microfluidics-based isolation methods. The selection of isolation techniques is depending up on the sources of exosomes [7]. The uptake mechanism of the exosomes from the recipient cells also varies based on the sources of exosomes, recipient cells, and other physiological conditions. The primary uptake mechanism includes phagocytosis, macropinocytosis, receptor-mediated uptake, fusion & direct release, endocytosis, caveolae, and/or lipid-raft-dependent endocytosis [8].

The biogenesis of exosomes takes place by various intracellular mechanisms before release of the matured exosomes which contains several outer membrane proteins and intracellular materials that tend to promote their physiological functions, ILV-Intraluminal Vesicles, MVB-Multivesicular Bodies, HSP-Heat Shock Proteins.

The characterization and quantification of exosomes help to identify the quantity, purity, and natural characteristics of exosomes. It divides into physical and chemical techniques such as Transmission electron microscopy (TEM) [9], Cryo-electron microscopy [1], Atomic force microscopy [1], Dynamic light scattering (DLS) [10], Nanoparticle tracking analysis (NTA) [11], Western blot [12], and Mass spectrometry [13]. Exosomes can be released by almost all eukaryotic cells and their cargos are likely to differ significantly depending on the origin cell type and present condition (e.g., transformed, differentiated, stimulated, and stressed). Non-hematopoietic cells such as astrocytes, adipocytes, and neurons also release exosomes. Body fluids such as urine, amniotic fluid [14], blood, saliva, serum, breast milk, ascites, cerebral fluid, and nasal discharge [15] are common sources of exosomes. Tumor cells can release a large number of exosomes which ultimately leads to promote angiogenesis, migration, proliferation, and support tumor progression [16]. Further, various stem cells [17], and immune cells derived exosomes [18,19] prevent the tumor cell invasion and treat the diseases. By transporting their contents, they play an important role in intercellular communication [20]. Exosome-mediated intercellular communication is involved not only in the control of normal physiological processes but also triggers various pathological conditions. Exosomes and their biologically active cargos could provide predictive information in various diseases, including chronic inflammation [21], cardiovascular and renal diseases [21,22] neurodegenerative diseases, lipid metabolic diseases, and cancers [20].

In this article, we took a board view to understand the exosome’s basic physiology and their functions in the systemic circulation. Also, we have tried to point out the current techniques to isolate, characterization, and quantification with their advantages and disadvantages. Further, we have included all the primary sources of exosomes and their biological and pathological functions with the challenges to overcome.

## Composition of Exosomes

Exosomes are thought to consist of many different components such as proteins (annexins, tetraspanins, heat shock proteins, etc.), lipids (sphingomyelins, glycosphingolipids, cholesterol), genetic materials (DNA, tRNA, miRNA, mRNA, small and long noncoding RNAs (lncRNA and sncRNA, respectively)), and small-molecule metabolites (ATP, amino acids, sugars, amides, etc.) [23]. Among the various components, lipids are the least studied, but they are considered an important component in the membranes of exosomes. The exosome membrane consists of different types of lipids such as sphingomyelin, cholesterol, glycosphingolipids, and phosphatidylserine. Lipids are involved in the construction of exosomal membranes as well as the synthesis and release of exosomes into the extracellular environment [23,24]. Exosomes are mostly composed of monounsaturated, polyunsaturated, and saturated fatty acids, while the lipid content of an exosome is dependent on the parent cells from which it is produced. Exosomes can carry a variety of bioactive lipids and enzymes involved in lipid metabolism. For instance, Mesenchymal stem cells (MSC)-derived exosomes contain fatty acids such as leukotrienes, arachidonic acid (AA), prostaglandins, phosphatidic acid, lysophosphatidylcholine (LPC), and docosahexaenoic acid (DHA). Exosome lipid metabolism enzymes can influence a recipient’s cell homeostasis [23,25,26]. Exosomes also contain a wide range of proteins on their surface [3]. The protein composition of the exosomes depends on the type of cell that secretes them. Proteins from endosomes, plasma membranes, and cytosol are found on most of the exosomes regardless of the cell type [6]. Different kinds of 14-3-3, heterotrimeric G proteins, and protein kinases are also found in exosomes, all of which are important in signal transduction during crucial physiological processes [23]. Exosomes can carry various nucleic acids including RNA, which has led to the discovery of exosomes as a modulator of intracellular communication and a component of many signaling pathways (Table 1) [23].

**TABLE 1:** Table depicting the various components of exosomes

Components	Localization	Function	References
**Proteins** Tetraspanins (CD63, CD9, CD81, CD82)	B cells, dendritic cells Enterocytes, Mastocytes T cells	Can be used as markers for exosome	[26]
Intercellular adhesion molecules (ICAM)	B cells, dendritic cells Mastocytes	Mediators of immune response	[27,28]
Heat shock proteins (HSP70, HSP70)	Tumors, Reticulocytes, dendritic cells, peripheral blood mononuclear cells,	Cell-cell communication, activation of immune cells, anti-inflammatory and antiplatelet response	[29,30]
**Nucleic acids** miRNA and mRNAs	Mesenchymal stem cells	Exosomal RNA helps in activating and inactivating the immune system	[31]

## Isolation Techniques of Exosomes

### Ultracentrifugation

For isolating exosomes, ultracentrifugation has typically been regarded as the most effective method. The primary advantage of this cutting-edge method is that it yields highly enriched extracellular vesicle (EV) fractions while enabling the collection of many other vesicle fractions. Due to its straightforward procedure and high yield, ultracentrifugation is widely employed [32]. It can separate particles with variable sedimentation rates and eliminate unwanted components during each centrifugation cycle by gradually increasing centrifugation speed or duration. It is worth noting that none of the methods for isolating exosomes is optimal. Each method has inherent limitations, which may be addressed by combining various techniques to produce the optimum desired characteristics. Exosomes’ productivity, purity, composition, and functioning depend on the method employed to separate and purify them. The widely used ultracentrifuge presently has poor adaptability, segmentation, and aggregation regarding exosome separation [32].

In cell culture samples, ultracentrifugation is a practical approach for isolating exosomes; however, samples from human sources, such as serum, include an intricate combination of various cell components, increasing the likelihood of co-sedimentation of unbound proteins, thus reducing the effectiveness of separation. Although centrifuging exosomes after passing them through a series of filters with nanometer pore sizes separates them from smaller protein contaminants, proving an effective method. However, it still risks contamination as the filtration pressure can cause larger microparticles to break into smaller vesicles [33]. The most popular method for removing cells and vesicles from culture fluid involves a systematic series of serial centrifugation steps, followed by concentration by ultracentrifugation and density gradient filtering. It is time-consuming, requires considerable effort, and relies on expensive equipment. Multiple commercially produced isolation kits are available for rapid, reproducible results [34,35]. These kits are proving to be more reliable when compared to other isolation techniques (Table 2).

**TABLE 2:** Principles of various isolation techniques and their properties

Methods	Principle	Duration	Advantages	Disadvantages	References
Ultracentrifugation	Size- and density-based separation under centrifugal force	2–4 h	Compatible for large volume samples, cheaper, fewer chances of contamination, higher yield of exosomes, protein and RNA molecules remain intact	Higher equipment cost, time-consuming, low purity	[36]
Precipitation	Based on differential solubility	1–12 h	Higher efficiency, easy to execute, concentrates samples, suitable for samples with large volumes	Higher probability of co-contaminants aggregates	[37]
Affinity based capture	Based on the interaction between antibodies with exosome membrane protein	4–20 h	Advantageous for isolation of specific subtypes of exosomes	Antibody specificity dependent, expensive method, lack of established markers, cross-reactivity of antibodies	[38]
Microfluidic technique	Based on selective antibody immobilization on a microfluidic device	1–2 h	Fast, economical, highly portable, low primary sample volume required	Nascent stage of development, lack of standardization and evaluation of clinical samples, lack of method validation	[38]

### Precipitation

The precipitation technique involves the aggregation of exosomes followed by precipitation with the help of polymers at a low centrifugation rate. For precipitation, polyethylene glycol (PEG) is widely used [39]. Pre-treating the samples with centrifugation or ultracentrifugation is essential to extract exosomes with no protein, lipoprotein, or other impurities [39]. Exosome precipitation kits are commercially available and some are even compatible with clinical samples such as body fluids, serum, urine, and CSF. Before precipitation, the samples must be freed of cells and cellular debris [40]. It is reasonably rapid and straightforward to execute, making it ideal for processing large numbers of samples at once. Unfortunately, the purity and recovery rate is poor, and the polymer may be hard to eradicate, interfering with further exosomal processing [41]. The co-precipitation of contaminants that are not exosomes, such as proteins and polymeric materials, is a severe disadvantage of polymer-based exosome precipitation. To counter such limitations, precipitation can be used with an immunoprecipitation technique or another exosome enrichment technique to isolate pure exosome fractions (Table 2) [42].

### Size exclusion

A size-based separation technique used in exosome isolation is size exclusion chromatography (SEC). Molecules with various hydrodynamic radii can be separated by SEC using a biofluid as the mobile phase and a porous gel filtration polymer as the stationary phase [43]. The size-based separation of macromolecules and particulate materials is achieved using a highly permeable stationary phase. Components achieve delayed elution in a sample with small hydrodynamic radii being able to flow through the pores. Exosomes and other components with high hydrodynamic radii are restricted from passing through the pores [43]. As a result, the separated exosomes exhibit improved purity, selectivity, and stability [42]. Compared to other exosome separation techniques, the usual processing time per sample is 20 min which is quicker than the other methods [44]. Poor yield is one of the limitations of this method. However, this may be overcome by using a large quantity of mobile phase [44].

Exosomes have also been isolated via exosome extraction by precipitation, which involves trapping exosomes in special filters called polymer nets and collecting them using basic, low-speed centrifugation at 1500× g. These techniques are straightforward and do not require sophisticated equipment, making it easier to implement them in clinical settings. This makes scaling the technique for more samples feasible [45]. Exosome isolation polymer must be made of harmless, inert components that do not provoke an immune response in both experimental and *in-vivo* conditions (Table 2) [46].

### Microfluidic technique

The separation process of the microfluidic technique is based on the physical and biochemical characteristics of specific exosome types. Separation techniques that utilize conventional microfluidics may be classified into immunoaffinity, sieving, and exosome separation employing porous materials. Microfluid-based isolation methods are still in their developmental stage. They will, nevertheless, be widely employed in diagnostics due to their benefits, such as minimal reagent volumes, extremely high purity of separated products, and rapid processing time. The immunoaffinity capture on a microfluidic device improves specificity and subtyping ability [47]. Porous silicon nanowire-on-micropillar structures were designed to identify exosomes from all other EVs and cellular debris [48], which trap exosomes with sizes ranging from 40 to 100 nm while filtering out proteins, other EVs, and cellular contaminants. Capture antibodies or beads that precisely target surface biomarkers of EV subpopulations are used in microfluidics-based immunoaffinity capture (Mf-IAC) (Table 2) [48].

## Characterization and Quantification of Exosomes

### Physical techniques

There are a wide variety of techniques used for the quantification of the exosomes, but a specific isolation technique must be employed before the quantification of exosomes [49]. The exosomes are subjected to quantification based on the size, shape, density, or presence of certain membrane proteins on the surface of the exosomes. There are various physical methods widely available to quantify the exosomes. The physical mehods include electron microscopy technique, dynamic light scaterring, and nanoparticle tracking analysis [15]. Electron microscopy including transmission electron microscopy (TEM) and scanning electron microscopy (SEM) is a popular and widely used technique in exosome analysis. The surface of the exosomes captured by SEM via passing the electron beam to the sample produces 3D image information as well as the composition. In TEM, the electrons do not interact with the sample particles rather it forms dark patches or shadows on the fluorescent screen that produce an image [50]. Both TEM and SEM reveal a comparable size distribution of the exosomes but TEM has a superior resolution to capture the image <1 nm in size [1].

Cryo-electron microscopy (Cryo-EM) examines the sample at a low temperature of −100°C [51,52]. Unlike SEM and TEM, it requires substantial sample fixation and staining procedures. Cryo-EM allows exosome examinations in the frozen sample to avoid dehydration and chemical fixation [1]. Another high-resolution imaging approach for the characterization of exosomes is atomic force microscopy (AFM) [53]. AFM involves passing a mechanical cantilever over a surface and measuring deflection to determine the existence and topography of surface features. Exosome samples can be adsorbed onto a mica holder and photographed after mild drying with AFM. Topographic AFM reveals a round morphology of isolated exosomes, whereas phase pictures reveal substructures thus showing variable constitutive elements (e.g., lipid, protein) [1].

Dynamic light scattering (DLS) is a technique that is used for exosome characterization. It determines the multiple physical attributes of exosomes in suspension by using the Brownian motion of the particles to estimate the size and concentration of the particles. The particle size measured by DLS involves fluctuations in the scattered light intensity, which will be used to determine the particle size of exosomes [54]. DLA is considered to be advantageous over other methods as it uses only little sample, and it is comparatively easy to handle with very few parameters required for optimization [55]. Nanoparticle tracking analysis (NTA), is an optical particle tracking method for determining particle concentration and size distribution [56]. The particles in the sample are illuminated by a light beam. A camera captures the path of each particle as it scatters light and moves in Brownian motion, allowing the mean velocity and diffusivity to be calculated. Unlike DLS, which measures bulk scattering, NTA measures individual particle scattering [57]. Particle size can be calculated by the Stokes-Einstein equation, where the diffusion coefficient depends on the Brownian movement of the particles. Particles will be made to interact with scattered laser light housed within a chamber. Light scattered by the particle will be captured by a microscope. The video recorded by the camera will be used to determine the movement of the particles using NTA software [1].

Size distribution and the particle concentration of the sample can be measured using a Tunable Resistive Pulse Sensing (TRPS) technique that is based on the coulter principle. This technique can be used to determine the size and concentration and it is used as an alternative to NTA [58]. It monitors transitory changes in ionic current caused by vesicle transport over a polyurethane membrane with a size-tunable nanopore. Akers et al. recently examined the performance of NTA and TRPS in detecting exosomes and microvesicles in patient CSF fluids. NTA consistently detected more exosomes than TRPS less than 150 nm in diameter, according to scientists [1]. Larger exosomes whose size is greater than 150 nm can be easily detected using TRPS instead of NTA (Table 3) [59].

**TABLE 3:** Advantages and disadvantages of various exosomes quantitative methods

Types	Quantification technique	Advantages	Disadvantages	References
Physical methods	Scanning electron microscopy (SEM)	The 3D structure and elemental composition of the sample can be studied	This may cause the loss of exosomes due to dehydration and embedding	[9]
	Transmission electron microscopy (TEM)	TEM has a superior resolution with capabilities to image	Labor intensive, expensive setup, low throughput	[9]
	Cryo-electron microscopy	No dehydration of the samples, DNA, RNA, lipids, etc. can be well differentiated	Time-consuming, expensive, cannot obtain the image of the tilted sample	[1]
	Atomic force microscopy	Do not require extensive sample preparation, provides a 3D image of the sample	The scanning speed is slow compared to SEM, Single scan image size, and labor-intensive	[1,11]
	Nanoparticle tracking analysis (NTA)	Does not rely upon the detection of specific markers and exosomes can be suspended in a wide variety of solutions	A large amount of sample is required (0.5 ml approx), only a limited range of particle measurement	[11]
Chemical methods	Western blot	Easy to use, possess the ability to detect exosomal proteins present outside and inside the exosomal membrane	Low yield, time-consuming	[12]
	Mass spectrometry	Provides high throughput quantitative and comparative EV proteomic analysis	Sensitivity is less compared to antibody-based techniques	[13]

### Chemical techniques

Chemical methods are widely available to quantify the exosomes including flow cytometry, western blotting, and mass spectrometry [15]. Flow cytometry is a technique used for characterizing single large particles, such as cells or larger micrometer-sized entities, using light scattering and fluorescence activation. However, traditional flow cytometry has limited resolution and sensitivity for detecting small particles with a diameter of less than 500 nm. It also has a strong optical background due to the presence of tiny particles (less than 200 nm) in the sheath fluids [60]. The detection range of the flow cytometer is much larger than the size of exosomes [61]. To observe exosomes through a flow cytometer, they must be immobilized on the bead surface by immunocapture technique or covalent attachment. The exosomal vesicles will be exposed to a fluorescently conjugated antibody against an antigen that is known to be expressed on the surface of the exosome after immobilization. Before flow cytometry, the exosomal vesicles coupled to the beads and the fluorescent antibody can be seen with an epifluorescent microscope (EPI). The sample then creates a fluorescent signal that is recognized as it travels past the flow cytometer’s laser [61].

Western blotting, commonly known as immunoblotting, is a popular protein analysis technique used in a variety of molecular biology fields. Western blotting is the most widely utilized approach for showing the presence of different target proteins allegedly related to exosomes [62]. Unlike western blotting, which only allows for small-scale quantification of specific proteins [63]. Purified exosome preparations are digested with enzymes and separated into peptides before being ionized and evaluated with a mass spectrometer. Mass spectrometry has become a crucial tool for proteomics research after being widely utilized to evaluate biological samples. The purification of exosomes and peptide fractionation was done before mass spectrometry analysis and is regarded as critical to identifying the vesicular proteins. This is usually accomplished in one of three ways such as SDS-PAGE [64], isoelectric-focusing-based fractionation, and two-dimensional liquid chromatography [65]. Direct quantification of exosomes is a very novel approach, and there is currently no consensus on the best method or how to evaluate data from multiple methods. Finally, these techniques will require more research to determine their reproducibility, dynamic range, sensitivity, specificity, and the protocols needed to sustain their performance across laboratories (Table 3) [54].

## Methods of Exosomes Uptake by Its Recipient Cells

Exosomes are small extracellular vesicles that can be taken up by recipient cells through various mechanisms, including phagocytosis, macropinocytosis, receptor-mediated uptake, fusion and direct release, endocytosis, and caveolae and/or lipid-raft dependent endocytosis. The uptake of exosomes through phagocytosis is an important mechanism for intercellular communication and the regulation of immune responses [66]. Understanding the mechanisms of exosome uptake and their biological effects can have important implications for the development of novel therapeutic strategies for the treatment of various diseases, including cancer and inflammatory disorders [66].

### Phagocytosis

**FIGURE 2:**
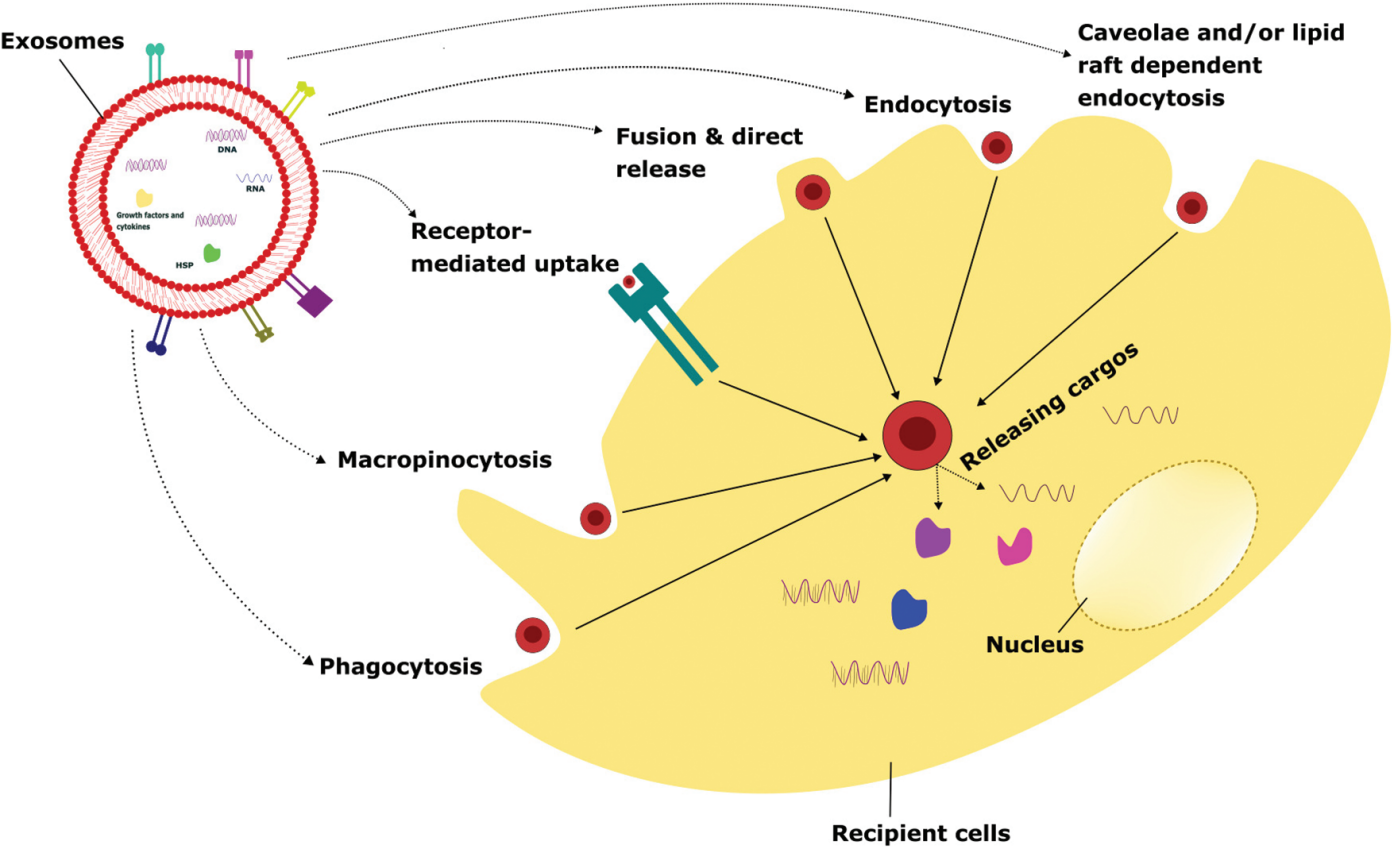
Various cellular uptake mechanisms of exosomes.

Phagocytosis is a process by which cells engulf and internalize particles, including microorganisms, cell debris, and extracellular vesicles, through specialized membrane structures called phagosomes [67]. Various types of phagocytosis processes take place to engulf the exosomes depending upon the nature and source of exosomes. It includes receptor-mediated phagocytosis where exosomes can be taken up by phagocytes through recognition and binding of specific receptors like CD44, integrins on their surface, and non-specific phagocytosis where phagocytes engulf exosomes based on their size, shape, and charge, and opsonin-mediated phagocytosis is a process that tags pathogens or other particles for recognition and uptake by phagocytes [68]. Exosomes can be opsonized with specific proteins, such as complement C3, which facilitates their recognition and uptake by phagocytes and co-culture with phagocytes where exosomes can also be taken up by phagocytes through co-culture. In this method, exosomes are added directly to the culture medium of phagocytes, allowing for their internalization through various phagocytic mechanisms. The uptake mechanism can influence the fate of exosomes and their biological effects on recipient cells. For instance, it has been shown that dendritic cells can take up exosomes derived from tumor cells by non-specific phagocytosis and present their antigens to T cells, leading to the activation of an anti-tumor immune response (Fig. 2) [68].

Exosomes internalize to the recipient cells via several mechanisms such as phagocytosis, macropinocytosis, receptor-mediated uptake, fusion & direct release, endocytosis, caveolae, and/or lipid raft-dependent endocytosis. Once the attachment of exosomes to recipient cells takes place, it releases the intracellular cargo that leads to altering the physiological functions depending on the exosomal particles and the cellular conditions.

### Macropinocytosis

Macropinocytosis is a process by which cells engulf large amounts of extracellular fluid and macromolecules by extending their membrane and forming macropinosomes. This mechanism is known to play a role in the uptake of exosomes by recipient cells [69]. The mechanism of exosome uptake involves the interaction between the exosome membrane and specific receptors or lipid rafts on the recipient cell membrane, which triggers the formation of macropinosomes and internalization of the exosome cargos. Studies have shown that exosomes can be taken up by cells through macropinocytosis, particularly in the case of cancer cells. For example, tumor-derived exosomes have been shown to induce macropinocytosis in recipient cells, allowing for their internalization and subsequent effects on cellular signaling and behavior [70]. Tumor-derived exosomes induced macropinocytosis in endothelial cells, which facilitated their uptake and led to increased angiogenesis, or the formation of new blood vessels [71].

Additionally, it can facilitate the uptake of nutrients, such as amino acids and glucose, by cancer cells and other rapidly dividing cells [71]. The ability of exosomes to induce macropinocytosis in recipient cells has important implications for intercellular communication, particularly in the context of cancer. Tumor-derived exosomes are internalized by recipient cells, including immune cells and stromal cells, through macropinocytosis, leading to changes in cellular signaling and behavior that can promote tumor growth and metastasis. Furthermore, the ability to target exosomes to specific cell types and trigger their uptake by macropinocytosis holds great promise for the development of novel targeted therapeutics (Fig. 2) [71].

### Receptor-mediated uptake

Exosome uptake by receptor involves the binding of exosomal ligands, such as surface proteins or lipids, to specific cell surface receptors on the recipient cell. This binding triggers the formation of coated pits, which are small invaginations in the plasma membrane that enclose the exosomes and transport them into the cytoplasm [72]. One of the best-studied examples of exosome uptake by receptor-mediated is the interaction between exosomal tetraspanins, such as CD63 or CD9, and the integrin receptor family. These interactions can trigger the formation of coated pits and the subsequent internalization of exosomes into recipient cells [73]. The uptake of exosomes by receptors is important for a wide range of physiological processes, including the regulation of immune responses, intercellular communication, and the transfer of genetic material between cells [73].

In addition, receptor-mediated uptake of exosomes may play an important role in the development of exosome-based therapies, such as targeted drug delivery or gene therapy [72,74]. The mechanisms of exosome uptake by receptors are complex and still being investigated. However, a better understanding of these processes could have important implications for the development of novel therapies that harness the therapeutic potential of exosomes [75]. In addition to the examples mentioned earlier, several other receptor-ligand interactions have been shown to mediate exosome uptake by receptor. For instance, exosomal proteins, such as annexins and galectins, have been shown to bind to specific receptors, such as low-density lipoprotein-related receptor protein 1 (LRP1) and T cell immunoglobulin and mucin domain 1 (TIM-1), respectively, on the surface of recipient cells, resulting in their internalization [73]. Furthermore, the uptake of exosomes by receptor can be modulated by a variety of factors, including the abundance and accessibility of exosomal ligands, the density and distribution of cell surface receptors, and the presence of competitive ligands or inhibitors (Fig. 2) [76]. The complex and context-dependent nature of exosome uptake by receptor underscores the need for further research to fully understand the mechanisms of this process and its potential applications in medicine and biotechnology.

### Fusion and direct release

Exosome uptake by recipient cells can also occur through the mechanisms of fusion and direct release. In the fusion mechanism, exosomes merge with the plasma membrane of recipient cells, allowing their contents to be released into the cytoplasm. In the direct release mechanism, exosomes are directly released into the cytoplasm of recipient cells through a process of membrane disruption [75,77]. The fusion mechanism of exosome uptake is thought to involve specific interactions between exosomal surface proteins and membrane receptors on the surface of recipient cells. One example of this mechanism is the interaction between exosomal tetraspanins and integrins on the surface of target cells. These interactions can trigger a cascade of intracellular signaling events that lead to the fusion of the exosome with the plasma membrane of the recipient cell [77]. The direct release mechanism of exosome uptake has been observed in certain cell types, including neurons and glial cells. In this mechanism, exosomes are released directly into the cytoplasm of recipient cells without the need for membrane fusion [75]. The direct release mechanism of exosome uptake may be particularly important in certain contexts, such as the transfer of genetic material between cells, where the delivery of intact exosomes may be required for the effective transfer of genetic information [74,75]. The mechanisms of fusion and direct release are thought to play important roles in the uptake of exosomes by recipient cells and may be particularly important for the transfer of specific types of exosomal cargo [72].

While the mechanisms of fusion and direct release are less well understood than other modes of exosome uptake, recent studies have shed light on some of the molecular and cellular processes involved in these processes. For example, it has been shown that exosome uptake by fusion is mediated by specific interactions between exosomal surface proteins, such as tetraspanins, and membrane receptors on the surface of recipient cells. These interactions can trigger a cascade of intracellular signaling events that lead to the fusion of the exosome with the plasma membrane of the recipient cell. Additionally, recent studies have suggested that exosome uptake by fusion may be influenced by the size and composition of the exosomes, as well as the physiological state of the recipient cells [72]. In the case of direct release, it is thought that exosomes are internalized into recipient cells through a process of endocytosis, which involves the formation of small vesicles around the exosomes and their subsequent release into the cytoplasm. Studies have suggested that direct release may be more efficient than other modes of exosome uptake in certain contexts, such as the transfer of genetic material between cells [74,77]. While the mechanisms of exosome uptake by fusion and direct release are still being investigated, and these processes play important roles in the transfer of exosomal cargo between cells (Fig. 2). Understanding the mechanisms underlying these processes may therefore have important implications for the development of novel exosome-based therapies, such as the use of engineered exosomes for targeted drug delivery or gene therapy.

### Endocytosis

Endocytosis is a process by which cells internalize extracellular materials, including exosomes, by forming vesicles derived from the plasma membrane. The process of endocytosis can be classified into different subtypes based on the size and composition of the vesicles formed. One such subtype, clathrin-mediated endocytosis, has been shown to play a role in the uptake of exosomes by recipient cells [72]. Clathrin-mediated endocytosis involves the formation of clathrin-coated pits on the plasma membrane, which bind to specific receptors and trigger the formation of clathrin-coated vesicles that contain the internalized material. Studies have shown that some exosomes can bind to specific receptors on the surface of recipient cells and trigger their internalization through clathrin-mediated endocytosis [72]. Exosomes derived from dendritic cells are internalized by recipient cells through clathrin-mediated endocytosis, which leads to their processing and presentation of exosome-derived antigens on MHC molecules for activation of T cells [78]. Similarly, exosomes derived from cancer cells are internalized by recipient cells through clathrin-mediated endocytosis, leading to the transfer of signaling molecules that promote tumor growth and metastasis. In addition to clathrin-mediated endocytosis, other subtypes of endocytosis, such as caveolin-mediated endocytosis have also been implicated in the uptake of exosomes by recipient cells. The choice of an endocytic pathway can depend on various factors, including the size and composition of the exosome, the cell type of the recipient, and the presence of specific receptors on the cell surface [76].

Some studies have also shown that the internalization of exosomes by endocytosis is a regulated process and can be influenced by various factors. For instance, the protein content of exosomes can affect their uptake by recipient cells, as different proteins on the surface of exosomes can interact with different receptors on the surface of recipient cells. In addition, the lipid composition of exosomes can also play a role in their internalization by recipient cells through endocytosis. Exosomes enriched in certain lipids, such as ceramide, sphingosine, and phosphatidylserine, are preferentially internalized by recipient cells through endocytosis [79]. Moreover, research has suggested that the process of endocytosis can be modulated by extracellular cues, such as growth factors and cytokines [79]. For example, it has been shown that treatment of recipient cells with certain growth factors, such as epidermal growth factor (EGF) and platelet-derived growth factor (PDGF), can enhance the internalization of exosomes through endocytosis. Similarly, the presence of inflammatory cytokines, such as TNF-α, can also increase the uptake of exosomes by recipient cells through endocytosis (Fig. 2) [72].

### Caveolae and/or lipid raft-dependent endocytosis

Exosome uptake by caveolae and/or lipid raft-dependent endocytosis is another mechanism by which exosomes can be internalized by recipient cells. Caveolae are small invaginations in the plasma membrane that are enriched in cholesterol and sphingolipids and are thought to play a role in the uptake of certain types of cargo, including exosomes [72]. Lipid raft-dependent endocytosis is a related process that involves the uptake of cargo through plasma membrane domains that are rich in cholesterol and glycosphingolipids, known as lipid rafts. Like caveolae, lipid rafts play a role in the uptake of exosomes. The exact mechanisms underlying exosome uptake by caveolae and/or lipid raft-dependent endocytosis are not fully understood. However, it has been suggested that exosomes may be internalized through the interaction of their membrane proteins and lipids with specific receptors on the surface of recipient cells, such as caveolin-1 and flotillin-1 [80]. Once internalized, exosomes can be transported to various intracellular compartments, such as endosomes, lysosomes, or the trans-Golgi network, where they can exert their biological effects on recipient cells.

By targeting specific receptors or signaling pathways involved in this process, it may be possible to enhance the uptake of exosomes by recipient cells and improve the efficacy of exosome-based therapies [72,81]. It has been shown that the glycosphingolipid GM3, which is enriched in lipid rafts, plays a role in the internalization of exosomes by recipient cells. In addition, several studies have suggested that certain membrane proteins, such as CD44 and integrins, may interact with specific receptors on the surface of recipient cells to promote exosome uptake [82]. In some cases, the uptake of exosomes by caveolae and/or lipid raft-dependent endocytosis may be mediated by endocytic pathways that are regulated by small GTPases, such as dynamin and RhoA [72]. For example, it has been shown that the RhoA pathway plays a role in the internalization of exosomes by cancer cells [72]. Moreover, multiple mechanisms of exosome uptake may likely be active simultaneously or sequentially, depending on the cellular context (Fig. 2) [82]. So, understanding the mechanisms underlying exosome uptake is important for the development of effective exosome-based therapies, which may require the use of strategies to enhance the uptake of exosomes by recipient cells.

## Various Biological Sources and the Physiological Functions of Exosomes

### Stem cells-derived exosomes

**FIGURE 3:**
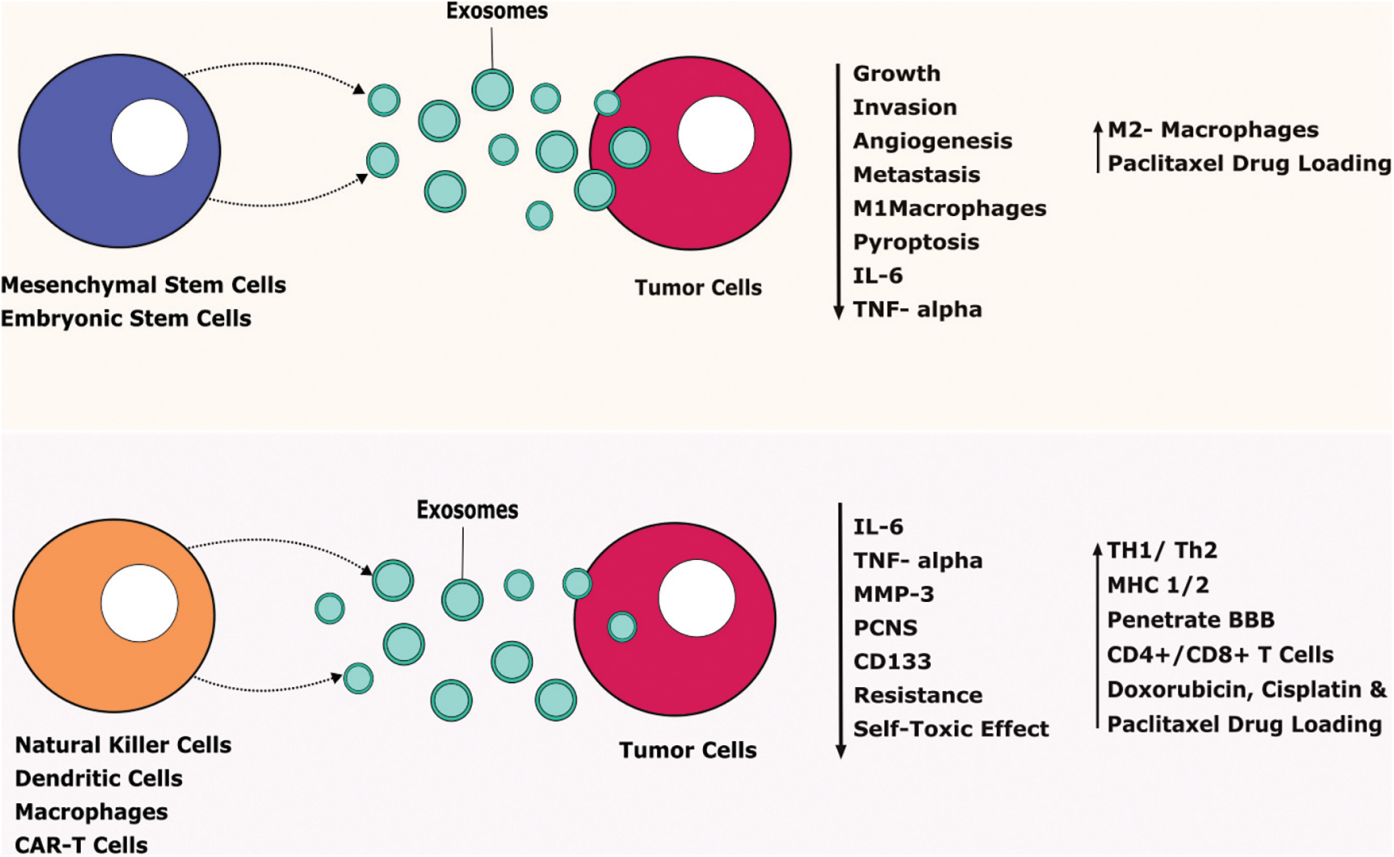
Therapeutic benefits of exosomes against tumor cells.

Mesenchymal stem cells-derived exosomes (MSC-EXOs) have been widely researched due to their unique characteristics such as being small in size (30–100 nm), tumor-homing properties, low immunogenicity, good penetration, biocompatibility, and long half-life [83]. It is considered the best choice to use as a delivery vehicle for animal studies. MSC-EXOs are round or cup-shaped with a density of 1.13–1.19 g/ml [83]. Engineered and non-engineered MSC-EXOs are broadly used to treat cancer. Non-engineered MSC-EXOs exert an anti-tumor action by inhibiting cancer growth, angiogenesis, invasion, and metastasis. Likewise, engineered MSC-EXOs showed a promising approach to use as a delivery vehicle to transport miRNA, proteins, and other small-molecule therapeutic agents [84]. MSC-EXOs contain various growth factors like hepatic growth factor (HGF), transforming growth factor-β (TGF-β), and anti-inflammatory agents including IL-1 receptor antagonist (IL-1Ra), and IL-10 which can efficiently modulate the immune system. Small molecules such as norepinephrine and N-methyl dopamine robustly enhanced the exosome production three-fold without modifying the capability of MSC-EXOs including macrophage polarization and inducing angiogenesis [85]. Human bone-marrow-derived MSC-EXOs (hBM-MSC-EXOs) attenuate the acute graft *vs*. host disease (aGVHD) in mice via regulating the T cells and dendritic cells (DCs) subpopulation function leading to the inhibitory inflammatory response. Also, these hBM-MSC-EXOs decrease the various inflammatory mediators such as IL-2, IFN-γ, and TNF-α and reduce the inflammatory condition both *in vitro* and *in vivo* (Fig. 3) [86].

Exosomes released from stem cells and immune cells secrete various agents that tend to prevent tumor progression. IL-Interleukin, TNF-Tumor-necrosis factor, MMP-Matrix Metalloproteinase, CD-Cluster of Differentiation, TH-T Helper Cells, MHC-Major Histocompatibility Complex, CAR T cells-Chimeric Antigen Receptor T cells, BBB-Blood Brain Barrier.

Adipose-derived stem cells (ADSCs) release more exosomes under hypoxic conditions. It has a wide range of biological functions including enhanced blood perfusion and suppression of inflammatory responses [87]. Hypoxia adipose-derived stem cells released exosomes support diabetic wound healing and inhibit inflammatory response by activating PI3K/AKT signaling pathways. It gives molecular insights into an alternative therapy to treat diabetic wounds [87]. Human ADSCs are a suitable source to use to promote tissue regeneration via secreting their paracrine mediators such as exosomes. hADSCs-derived exosomes (hADSCs-EXOs) supported the neural differentiation and cell viability in the PC-12 cells. Human embryonic stem cells-derived exosomes (hESC-EXOs) release large-scale exosomes and provide abundant sources for clinical application to use as a carrier for various chemotherapeutic agents like paclitaxel to treat GBM [88]. Doxorubicin is an anticancer agent, unfortunately, it causes muscle and cardiac toxicity due to increased inflammatory and oxidative stress. ESC-EXOs reverse the doxorubicin-induced pyroptotic-inflammatory condition on muscle cells such as Sol8 cells [17]. Moreover, ESC-EXOs significantly upregulate anti-inflammatory M2 macrophages and inhibit M1 macrophages that decrease inflammasome and counteract pyroptosis to improve muscle function. ESC-EXOs alleviate inflammatory mediators including IL-6 and TNF-α to support muscular function (Fig. 3) [89].

### Tumor cells-derived exosomes

Tumor detection at the early stage has been considered a vital strategy for tumor control. Notably, Tumor-derived exosomes (TDE) contain various cellular proteins that turn them into attractive biomarkers for diagnosis, cancer development, metastasis, and monitoring drug efficacy [90]. Typically, TDEs are larger compared to non-cancer cells derived exosomes due to being highly heterogenous and containing various overexpressed genes and proteins. For this reason, TDEs are referred to as oncosomes (100–400 nm) or even larger (1–10 µm) in size that carries abnormal macromolecules including oncoproteins [16]. TDE alters the biological characteristics of tumor cells to promote metastasis and growth [91]. TDE is taken up by the recipients’ cells via direct fusion into the membrane in different aspects such as lipid raft, clathrin-dependent endocytosis, phagocytosis, macropinocytosis, and caveolae [92]. Chronic myelogenous leukemia (CML) is a hematological disorder that occurs in adults with an incidence of 1 to 2 cases per 100,000 population [93]. According to the report, CML cells such as K562-derived exosomes induce more immunosuppressive and support tumor progression [94]. Generally, tumor-derived exosomes show detrimental effects on the immune system by impairing T cell immunity and suppressing the innate immune system leading to an increased tumor progression [95]. For instance, CML-derived exosomes suppress the cytotoxic T cells and elevate various genes including FOXP3, IL-17, and IL-6 that may stimulate the tumor-favorable T cells such as regulatory T cells [93].

**FIGURE 4:**
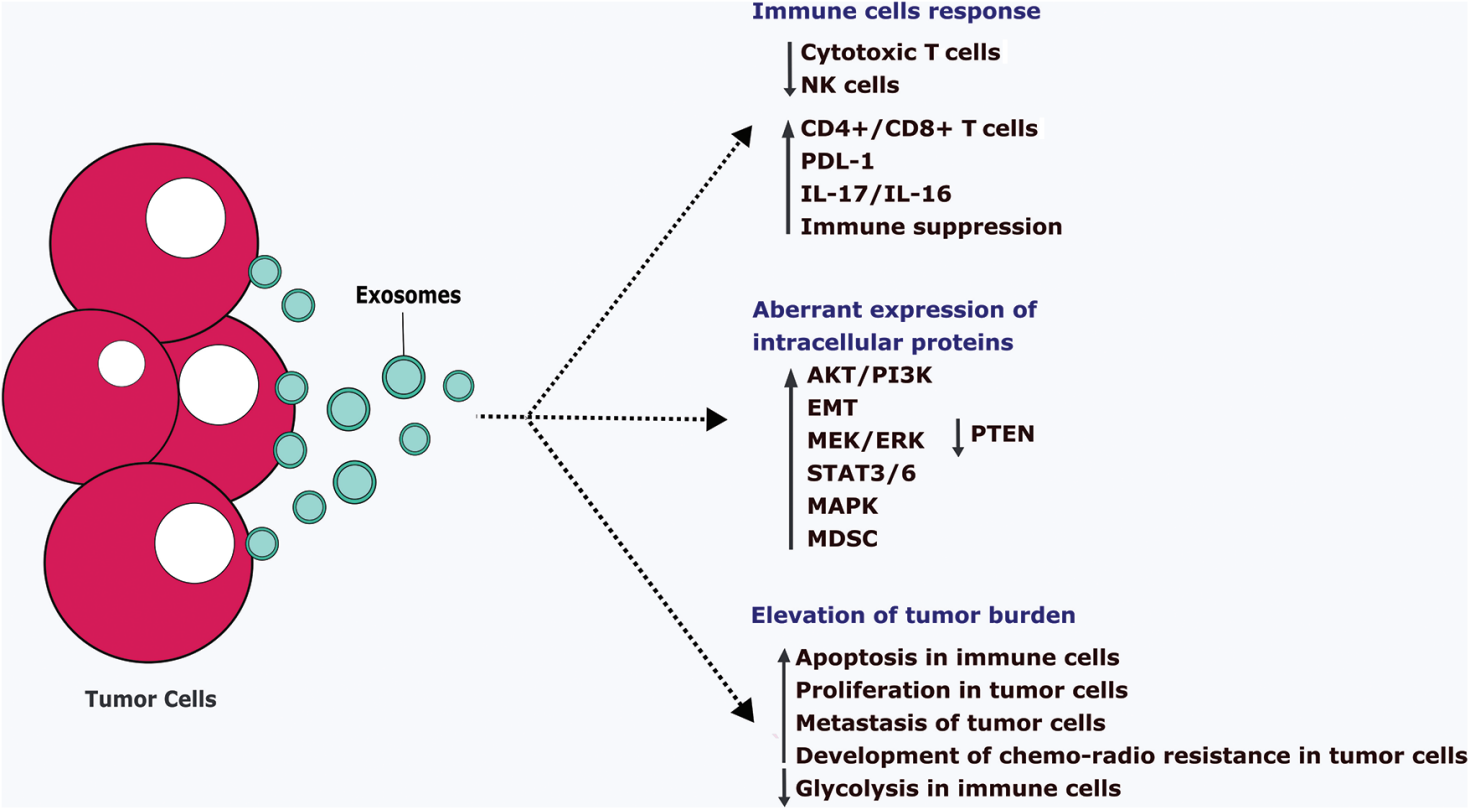
Pathological action of tumor cells-derived exosomes.

TDE potentially impede the anti-tumor immune response via their immunosuppressive cargo. TDE suppresses the anti-tumor immune response either by receptor-ligand interactions or internalization by the target cells [96]. TDEs harbor numerous membrane-bound proteins (PD-L1) that directly inhibit the NK cells and CD8+ T cells. Ning et al. found that DC treatment with TDE significantly inhibits the maturation of DC [97]. Also, TDE directly targets the DC cells which are the primary and effective regulator of APC leading to the development of tolerance resulting in it strongly promoting the tumor-supportive cells including myeloid-derived supportive cells (MDSC) [96]. TDE could trigger the anti-tumor environment into a pro-tumor environment by inducing tumor-associated stromal cells and EMT that impose therapeutic resistance and metastasis [98]. TDE promotes lung cancer through various mechanisms such as boosting EMT that induce angiogenesis and cause immune escape. Compared to normal cells, tumor cells secrete more exosomes that support invasion and metastasis [99]. Studies reported that TDE provides a favorable microenvironment such as increasing invasion, immunosuppression, and development of chemo-radiotherapy resistance for lung cancer progression [99,100]. For instance, exosomes derived from lung cancer express high PD-L1, reduce T cell action and cytokine production, and induce apoptosis in CD8+ T cells leading to immune escape [99]. Likewise, exosomes derived from bladder cancer cells such as MB-49 induce macrophage M2 polarization via activating AKT/STAT3/6 and inhibiting the PTEN signaling pathway leading to an immunosuppressive tumor microenvironment that facilitates tumor progression (Fig. 4) [101].

Tumor cells-derived exosomes (TDE) ultimately suppress the anti-tumor immune cells such as cytotoxic T cell and NK cells and tend to aberrantly express the intracellular proteins. It leads to the downregulation of the immune system via increasing apoptosis, reducing the metabolic pathways. Also, TDE stimulates the proliferation, metastasis, and development of chemo-radio therapy resistance in the tumor cells which ultimately supports the growth of the tumor cells. IL-Interleukins, EMT-Epithelial-mesenchymal transition, PTEN-Phosphatase and tensin homolog, ERK-Extracellular signal regulated-kinase, STAT-Signal transducer and activator of transcription, MAPK-Mitogen-activated protein kinase, MDSC-Myeloid-Derived Suppressor Cells, PI3K-Phosphatidylinositol 3-kinase, PDL1-Programmed Cell Death Protein 1, NK-Natural killer cells, CD-Clusters of differentiation.

Hypoxic conditions tend to release a large number of exosomes containing various oncogenic nucleic acids and proteins that support tumor progression. For instance, hypoxic TDE contains rich miR-31-5p expression and it enhances lung adenocarcinoma metastasis via negatively regulating special AT-rich sequence binding protein-2 (SATB2)-reversed EMT and promoting MEK/ERK signaling pathway [102]. Also, Li et al. reported that hypoxia ultimately increases the exosomal miR-21 and enhances the migration and invasion of oral squamous cell carcinoma (OSCC) via HIF-1α and HIF-2α dependent manner [103]. Exosomes are the vital mediators to reshape the tumor microenvironments in breast cancer (BC) that supports invasive behavior and metastasis. BC-derived exosomes could trigger the proliferation and metastasis that leads to the enhancement of the oncogenic phenotype [95]. Piao et al. reported that BC-derived exosomes can stimulate the macrophage polarization that provides the favorable condition for metastasis of triple-negative breast cancer [95]. Also, BC-derived exosomes transmit oncogenes pro-angiogenic signals that enhance the tumor vascularization, invasion, and stromal remodeling which leads to rendering cancer cells more aggressive and prone to metastasis. It also increases the myeloid-derived suppressor cell accumulation that suppresses T cell proliferation and NK cells leading to the weakened anti-tumor immune response in the premetastatic niche [95]. Qu et al. reported that gastric cancer cell-derived exosomes trigger intracellular signaling pathways such as MAPK and PI3K/Akt/mTOR that lead to promoting tumor growth (Fig. 4) [104]. The development of resistance in the cancer cells against the treatment is a major challenge to overcome. Bach et al. reported that TDE causes acquired resistance via transferring nucleic acids such as miRNA to the cancer cells [105]. TDE transfer ATP-binding cassette (ABC), also referred to as multidrug resistance (MDR) [19] to the recipient cells in various malignancies including breast cancer [106], prostate cancer [107], osteosarcoma [108], and melanoma [109] lead to promote the drug resistance.

Exosomes released by various sources promote the pathogenesis and progression of multiple myeloma (MM) via transferring its cargo such as proteins, cytokines, non-coding RNA, and lipids. For instance, exosomes containing miRNA induce cytokines and alter their productions that favor the growth of MM cells. Murine MM cells released exosomes transport diverse angiogenic-inducing proteins including HIF-1 and pathways such as STAT3, p53, and c-Jun N-terminal kinase that promote angiogenesis more in hypoxic conditions. MM-released exosomes cause the immunosuppressive action that leads to decreased immunosurveillance against tumor growth [110]. Also, MM-derived exosomes discharge a large number of ectoenzymes such as CD38 that transform nucleotides to adenosine leading to immunosuppression. Further, adenosine binds to the purinergic P2 receptor in the immune cells membrane which causes an insufficient response to the DC, T cells, and NK cells [111]. MM-derived exosomes cause drug resistance such as bortezomib via transferring non-coding RNA including miR-16, miR-17, miR-15a, and miR-20a [112] and acid sphingomyelinase (ASM) [113]. In cancer, TDEs are considered a noninvasive biomarker, especially those who underwent immune therapy. Ineffective antitumor immune response has been found due to TDE and may cause tumor immune escape, resistance, and unresponsiveness to the therapies [114]. Studies reported that coincubation of TDE and DC caused rapid internalization that leads to the downregulation of CD86 and CD80 on the surface of DC and inhibits DC maturation [115].

Furthermore, Ning et al. reported that lung carcinoma or 4T1 breast cancer-derived exosomes inhibit the maturation and migration of mouse bone marrow-derived DC indirectly promoting the immunosuppression and PD-L1 expression. Also, it drastically decreased CD4+ IFN-γ^+^ Th1 differentiation and increased the expression of regulatory T cells [116]. TDE bearing PD-L1 on their surface ultimately interacts with the T cells leading to the stronger inhibition of T cells resulting in inducing tumorigenicity and immunosuppression. PD-L1 expression in the TDE is higher in various cancer and it worsens the prognosis of patients [91]. TDE-bearing PD-L1 is considered a potential biomarker in various cancer such as melanoma [117], NSCLC [118], basal-like breast cancer (BLBC) [119], and pancreatic cancer [120]. Glioma cell-derived TDE turns the macrophages such as M0, M1, or M2 into highly suppressive tumor-associated macrophages (TAM) leading to upregulating arginase 1, and IL-10 that cause immune suppression and promote the glioblastoma cell growth (Fig. 4**)** [121]. TDE modulates the immune response and increases the programmed cell death (PD-1) protein that alleviates the glycolysis pathways in the immune cells [122]. Tumor cells tend to produce more exosomes because they can alleviate the immune cells’ response against the tumor cells via altering the metabolic pathways including glycolysis [123]. Acute myeloid leukemia (AML) released exosomes potentially induce the glycolysis process in the human umbilical vein endothelial cells (HUVEC) that leading to increased resistance and proliferation to the tumor cells [124].

### Immune cells derived exosomes

Biogenesis and other following steps like the release and uptake of immune cells-derived exosomes are regulated by intracellular proteins and extracellular stimuli. It mediates a cross-talk between the innate and adaptive immune systems and regulates cancer metastasis and progression. The immune cells-derived exosomes have dichotomous roles in cancer either by inducing suppressive or activating immune responses. So, immune cell-derived exosomes have a diverse role in tumor diagnosis and immunotherapy. It could potentially be developed for drug transportation and vaccine development against tumor cells [124]. Human dendritic cells released exosomes are the potent stimulator of NK cells that may occur via IL-15Rα or TNF superfamily ligands [125,126]. Dendritic cells-derived exosomes migrate to tumor cells and present antigens directly or indirectly to CD4+ and CD8+ T cells that induce an immune response [18]. This process can take place in several mechanisms such as direct restimulation of activated T cells [127], a cross-dressing method that indirectly activates antigen presentation to T cells, and tumor cells internalization that converts tumor cells into potent immunologic targets for effector immune response [127]. T cells and CD8+ T cells derived exosomes contain cytotoxic molecules and exhibit potent anti-tumor actions [19].

DC can secrete exosomes in a variety of sizes including small exosomes that can induce Th1 cytokine and large exosomes that stimulate Th2 cytokine secretion [18,128]. DC-derived exosomes additionally carry various antigen and co-stimulatory molecules including MHC I/II, CD4+, and CD8+ T cells that show more potent anti-cancer efficacy than DC vaccines in pre-clinical studies [129]. The study reported that DC-derived exosomes promote tumor-specific CD8+ T cells and a single injection of DC exosome administration achieved a better anti-cancer efficacy than DC alone. DC-derived exosomes achieved only limited in the clinical trials and that might be due to the usage of peptide-loaded DC-derived exosomes from monocyte-derived exosomes [129,130]. However, DC-derived exosomes have several advantages over DC including long shelf life, safety, fewer adverse events, reach and delivery of the cargoes at the targeted sites [131], and overcoming resistance to activate T cells and NK cells rapidly [132,133].

The rate of exosome secretion differs from the immune cells depending on its intracellular proteins and extracellular stimuli [134,135]. For instance, B cells and DCs release large amounts of exosomes upon radiation exposure, T cell interaction, or senescence induction [136]. Likewise, mature DCs secrete more exosomes than immature exosomes [19]. Also, only mature DCs derived exosomes could promote the interchange of MHC complexes between DCs that activate CD4+ T cells [137]. Compared to DCs, DCs-derived exosomes have several advantages that exert anti-cancer efficacy via promoting immune response and suppressing tumor progression [19]. Exosomes transfer information from donor cells to other immune cells under certain treatments. For instance, NK cells previously exposed to neuroblastoma (NB) cells secrete exosomes containing NK cell receptors such as CD56, KIR2DL2, and NKG2D. These exosomes educate NK cells to become more cytotoxic against NB cells [138].

Several immune cells including NK cells derived exosomes activate an anticancer immune response that contributes to tumor cell clearance. NK cells-derived exosomes carry FasL in the membrane and show a strong anti-cancer effect by clearing Fas^+^ tumor cells [139]. Likewise, NK-92 and NK-derived exosomes enriched with miR-186 exert cytotoxic effects against melanoma and NB cells via acting on the TNF-α and MYCN signaling pathways [140,141]. NK cells-derived exosomes show anti-cancer efficacy against the murine mammary tumor model. It decreases the tumorigenesis marker such as matrix metallopeptidase 3 (MMP-3), interleukin-6 (IL-6), tumor necrosis factor-α (TNF-α), and proliferating cell nuclear antigen (PCNS), and stem cell markers including CD133 leading to reducing tumor size of REM134 canine mammary carcinoma [142]. Enomoto et al. reported that exosomes derived from NK-92 cells penetrate the target cells via macropinocytosis and co-stimulated the cytokines including IL-15 and IL-21 showing potent cytotoxic efficacy against tumor cells [143]. M2 macrophage-derived exosomes enriched lncRNA such as AGAP2/AS1 enhances radiotherapy immunity against lung cancer via elevating Neurogenic locus notch homolog protein 2 (NOTCH2) and reducing miR-296. Further, exosomal AGAP2/AS1 has been considered a diagnostic biomarker for non-small cell lung cancer (NSCLC) [144].

Seo et al. reported that CD8+ T cells from healthy mice-derived exosomes induce apoptosis of mesenchymal tumor stromal cells in tumor-bearing animals that lead to alleviating tumor growth [145]. Mouse macrophage-derived exosomes were hybridized with synthetic liposomes to engineer hybrid exosomes (HE). HE-loaded doxorubicin potentially enhanced the toxicity against breast tumor cells and also can release the drug in acidic conditions, which benefits the delivery of the drug in the acidic tumor environment [146]. Exosomes regulate the immune system via transferring antigenic peptides, gene expression regulation by exosomal miRNA, inducing various signals by exosome surface ligands such as FasL, and induction of cGAS-STING transmission signal by DNA in recipient cells [147]. The functions of exosomes reflect their parent cells and their phenotypes, so the physiological action may vary depending on its source [148,149]. However, macrophage-derived exosomes exhibit various benefits such as penetrating BBB and preventing neurological diseases, attenuating toxic effects, carrying anti-cancer agents like cisplatin and paclitaxel to target the cancer cells, and preventing drug resistance (Fig. 3) [150–152]. Studies reported that CAR T cells-derived exosomes reduce the self-toxic effect and can cross the BBB and blood-tumor barrier. CAR T cells derived exosomes exert a high level of cytotoxic molecules and drug loading and delivery capacity via penetrating extracellular matrix that inhibits tumor growth with fewer side effects [153,154].

### Other body fluids

Exosomes are secreted by the cell of all organs and are found in various body fluids such as breast milk, serum, urine, amniotic fluid, etc., [155]. Levels of the exosomes derived from the serum of patients suffering from hepatocellular carcinoma (HCC) are found to be high. Hence the exosomes are considered biomarkers for the HCC [156,157]. Exosomes derived from the serum of patients suffering from pancreatic cancer proved to be involved in cell proliferation, migration, and epithelial-mesenchymal transition (EMT) thus supporting the role of exosomes in metastasis [154]. Exosomes can also be used as a biomarker in the diagnosis of glioma. miR-3184 in cerebrospinal fluid (CSF)-derived exosomes was enhanced in the development of glioma and its progression. The level of miR-3184 was found to be downregulated in glioma patients after the resurrection of the tumor [158]. Proteomic sequencing of amniotic fluid-derived exosomes also contributes to the enrichment of inflammatory markers in term and preterm pregnancies that are associated with term and preterm parturition [159]. When compared to normal tissues, the expression of PD-1 on CD8+ T cells in tumor tissues is significantly higher. Patients with overexpressed PD-1 had lower overall survival (OS) and disease-free survival (DFS) rates. Serum-derived exosomes increase PD-1 expression on CD8+ T cells and decrease their ability to kill, and secretory process [160]. Serum-derived exosomes contain numerous cancer-specific markers. But no study has attempted to analyze the function of serum-derived exosomes working as antigens to engage T cells and cause HCC inhibition [161].

Milk-derived exosomes (MDEs) serve as messengers between cells. Lipids, proteins, mRNAs, DNAs, and microRNAs are found in MDEs [162]. MDEs can be isolated from various sources including bovine colostrum, pig milk, rat milk, goat milk, wallaby milk, and human breast milk [163–165]. The most widely used technique to isolate exosomes from milk is ultracentrifugation with ultrafiltration to purify the exosomes by using membrane filters. It is primarily obtained by precipitation methods using polyethylene glycol and then using low-speed centrifugation or filtration, the precipitate is isolated [166]. Studies in both *in vivo* and *in vitro* have shown that milk-derived exosomes are absorbed by mammals and showed biological activity. Cells can absorb milk-derived exosomes miRNA, which can also cross the intestinal barrier and reach the bloodstream. Exosomes generated from milk and their RNA cargo may circulate in the blood and build up in numerous tissues [167–169]. An infant’s immune system development largely depends on breast milk. Milk-derived exosomes may provide strong immune regulation actions due to the presence of immunoregulatory miRNAs. Exosomes from human milk suppress autologous or allogeneic peripheral blood monocytes from producing pro-inflammatory cytokines like interleukin-2 (IL-2) and interferon (IFN). These exosomes also raised the number of regulatory T cells- Foxp3+ CD4+ CD25+ [170]. Exosomes from pig milk considerably decreased the toxic effect of deoxynivalenol to prevent intestinal epithelial cells in mice [171].

The potential of exosomes as therapeutics is currently being actively investigated. Milk-derived exosomes have been demonstrated to be a cutting-edge and efficient therapy for necrotizing enterocolitis (NEC), a dangerous condition that puts premature infants’ lives in danger. Studies have shown that human milk-derived exosomes exhibited antiapoptotic and pro-proliferative effects, lowering the prevalence and reducing the symptoms of NEC using animal models for the disease [172]. Medical specialists and clinicians can create safe and targeted therapies for treating a variety of diseases using milk exosomes, which can also provide biocompatible carriers for the transport of drugs. Exosomes produced from tear films have the potential to serve as sensitive, diagnostic biomarkers for the detection of prostate and breast cancer as well as primary Sjogren syndrome, primary open-angle glaucoma, and thyroid eye disease [173]. Tear exosomes possessed a much-increased concentration of exosome markers (CD9) than serum exosomes (CD63). Exosomes are more active and produce more inflammatory cytokines because of an increase in tear film (TF) number, which could induce tissue remodeling and inflammation. Increased exosome production and the expression of proteins in exosomes from TF may indicate pathogenic thyroid eye disease (TED) Exosomes are released by nearly all cells, hence cells from the lacrimal gland, corneal and conjunctival fibroblasts, and epithelial cells may be the sources of exosomes in tear fluid [174].

Increased amounts of cytokines such as IL-6, IL-1, IL-17A, IL-13, TNF-, IL-18, and RANTES were found in TF from TED patients [175]. Exosomes are an appealing diagnostic and treatment option for immunologically mediated eye disorders since they regulate multiple components of the immune system. However, the study of exosomes in the eye is still in its infancy and needs further research into the precise biological mechanisms and therapeutic possibilities of exosomes in ocular illnesses [176]. Exosomes in human urine originate from each type of urinary tract cell, beginning with the kidney and glomerular podocytes. Abundant and non-invasive urinary exosomes are available and provide an accurate representation of all urinary tract cell types. Exosomes that collect in urine are a rich source of biomarkers and a potential non-invasive diagnostic tool for renal illness [177]. EGF-like repeats and discoidin domains 3 (EDIL-3) promote angiogenesis and urothelial and endothelial cell migration. Exosomes isolated from the urine of individuals with high-grade bladder cancer consistently had noticeably greater concentrations of EDIL-3 [177]. The size range of salivary exosomes is between 30 and 100 nm, and they have abundant CD81, CD9, and CD63 immunoreactivity on their surface. Xerostomia and salivary gland dysfunction in diabetic rats can be improved by salivary exosomes’ via their anti-inflammatory and antioxidant characteristics [178].

## Diverse Therapeutic Advantages of Exosomes

The therapeutic functions of exosomes can be understood based on their three specific functions such as exosomes as transporters of drugs into targeted cancer cells and enhancing their effectiveness in treating cancer, exosomes as promoters of apoptosis, anti-angiogenesis, chemosensitivity, and immunotherapy, and act as diagnostic tools for detection of diseases at early stages [15].

Due to their high tolerance, highly invasive, long half-life, non-toxic, and non-immunogenic properties, exosomes promote the delivery of various drugs including adriamycin and paclitaxel to treat cancer [179–183]. The capacity of exosomes to identify the cancerous cells and infiltrate them makes drug delivery more efficient and targeted. Exosomes are also capable of increasing the rate of uptake of drugs by the cancer cells, reducing the side effects of different chemotherapeutic drugs like cisplatin or paclitaxel on healthy cells [184–186]. Their aqueous core and lipid bilayers help in encapsulating hydrophilic as well as lipophilic drugs, improving the solubility, stability, and bioavailability of various miRNAs and chemotherapeutic drugs. Exosomes are also functional in overcoming any pH-gradient-related challenges during the transportation of these drugs [182,184]. Certain exosomes are also capable of crossing the BBB, like those derived from macrophages and neutrophils, improving their efficacy in delivering drugs to the targeted sites in the brain [184–186]. Several studies have revealed that cisplatin-loaded M1 mononuclear macrophage exosomes are the potential tools to deliver chemotherapeutics, even to those cells that are resistant to the drugs [184]. Exosomes have enhanced the anti-tumor outcomes of several drugs such as paclitaxel and doxorubicin. Exosomes carrying paclitaxel have been effective in treating lung, pancreatic, and prostate cancer while exosomes loaded with doxorubicin have been giving better results in shrinking the tumor size and in treating breast cancer [179]. The exosomes generated by astrocytes transport PTEN-targeting miRNAs to prevent the expression of phosphatase and tensin homolog (PTEN) in cancer cells [179]. The molecular composition and immunogenicity of exosomes enhance the transportation of various nucleic acids, proteins, and genes and causes inhibition of angiogenesis and metastasis in the tumor cells [185,187].

Exosomes have been considered potential vaccines for treating cancer because of their capacity to transport antigens and MHC-peptide complexes and their role in enhancing immune responses [184]. Exosomes generated by dendritic cells (known to be immune system modulators) are most effective in immunotherapy because they trigger an immune response by presenting tumor antigens and enhance the effectiveness of the immune system [183,186–188]. Studies have proved that these exosomes carrying tumor antigens can stimulate CD4+ and CD8+ T cells, producing adaptive immune responses and anti-tumor T cell responses, leading to the shrinkage of the tumor cells [184,185,187]. A study conducted by Yufeng Xie et al. found that the modified exosomes produced from myeloma cells generated antitumor immunity by enhancing cell responses of type 1 T helper (Th1) and P1A-specific cytotoxic T lymphocytes when administered intravenously [186]. Exosomes produced from cells infected with the virus are capable of triggering an antiviral interferon (IFN) response, which promotes the development of vaccines for cancer. Exosomes derived from NK cells are found to produce antitumor effects against aggressive melanoma [181,183]. Modified exosomes that carry Tumor Necrosis Factor (TNF)-related Apoptosis-inducing Ligand (TRAIL) induce apoptosis in cancer cells, thereby decreasing tumor growth [179,182,186]. This anti-cancer protein (TRAIL) also diminishes the TRAIL resistance that is found in many different cancers like lung cancer, renal cancer, neuroblastoma, and so on [180]. Exosomes loaded with modified survivin are also found to be effective in inducing cell death in pancreatic adenocarcinoma cells [179,186]. Exosomes are also used to transport miRNAs and siRNAs, which are responsible for promoting chemosensitivity and inducing the death of cancer cells [179,186].

Exosomes are also effective biomarkers that help in the early detection of cancers and in preventing their growth [186]. For instance, elevated levels of exosomal miR-21 reveal the chances of glioblastoma, pancreatic, colorectal, liver, ovarian, and esophageal cancers, while the reduced level of tumor suppressor miRNAs, like miR-146a and miR-34a indicate the presence of liver, breast, and hematologic malignancies [189]. The polarizing function of exosomes has helped to develop therapeutic exosomes to improve anti-tumor immune responses [189]. The ability of exosomes to protect nucleic acids from disintegration and their high stability in circulation makes the liquid biopsy easier and aids in the diagnosis and prognosis [181]. The use of exosomes in diagnosis has also reduced the need for surgical examination of brain tissues in glioblastoma and other tumors [184]. The alterations in the contents of exosomes provide information for the diagnosis of cancers, thereby improving the therapeutic outcomes and increasing survival rates. Tetraspanins like CD9 or CD63 and the circulating tumor DNA (ctDNA) can be used to determine the progression of cancer and prognosis [186]. Studies have revealed that DNAs found in serum exosomes can be useful in detecting any mutations like KRAS and TP53 in patients with pancreatic cancer [189].

## Challenges Associated with Exosomes

Improving the loading efficiency and target specificity, without changing the physical properties or composition of the exosomes is the greatest challenge in the application of exosomes in cancer treatment [190]. The sufficient production of exosomes, that are pure and of high quality to produce an effective treatment response is one of the major technical tasks [191,192]. Lower production rates of exosomes limit their application at broader levels [193]. Cellular aging resulting from exhaustion can reduce the rate of production of exosomes and affect their functional capacity [194]. Recognizing optimal conditions necessary for exosome production can also be a difficult task [195]. To improve the efficiency and reliability of exosomes in delivering drugs, there needs to be mass production of reproducible exosomes, which requires standardized and effective manufacturing procedures, that are not yet developed [196,197].

Another greatest challenge is the lack of standardized techniques used to isolate and purify them [197–201]. The most commonly used techniques for the process of isolation of exosomes are ultracentrifugation, precipitation, size exclusion, affinity-based capture, microfluidic technique, and immunoprecipitation. But these techniques have their limitations and are found to be costly, time-consuming, laborious, and often result in contamination of exosomes that leads to affecting their functioning and efficiency in the treatment process [194,197,202,203]. Isolation of exosomes from biological fluids can be very difficult, since there is a higher chance of having size overlaps with other components like the lipoprotein, or even with other EVs, due to insufficient biomarkers [194,197]. The smaller size of exosomes makes it challenging to obtain clear images and differentiate them from other cell structures [197]. The lack of separation methods for separating free nucleic acids from loaded exosomes can also give inaccurate results [196].

Additional challenges associated with exosomes are related to the identification and quantification of exosomes. These procedures require costly equipment and often provide inaccurate results [203]. Exosomes can originate from a variety of cell types in the human body and it is necessary to have effective characterization procedures to use exosomes as drug delivery systems [197]. Exosomes derived from different cell types consist of different contents and can exhibit different functions [196,202]. Sometimes, even the exosomes that originated from the same category of donor cells can show variable properties, affecting their reliability [197]. The heterogeneous nature of exosomes, including the differences in sizes, constitution, roles, and cellular origin, makes it difficult to identify the delivery contents and results in mixed-size distribution [197,200]. This heterogeneity and the impurities found in the composition of exosomes interfere with the use of exosomes as diagnostic tools [200,204]. Changes in parent cell types, culture conditions, and insufficient isolation and characterization methods can result in an overlap between the chemical and physical properties of exosomes [200]. The process of isolation of exosomes itself can produce changes in their characteristics, like size and shape, so it would be beneficial to note the time between their isolation and characterization to obtain accurate results [197,202]. The differences in origin and in the techniques used to isolate and purify result in exosomes exhibiting variable properties. Therefore, the techniques used for isolation and purification should be standardized, rapidly acting, and cost-effective [202].

Inefficient loading and delivery of drugs can also be another challenge associated with exosomes [205]. During the production phase, exosomes largely consist of contents from parent cells, leaving only very little space for loading the therapeutic drugs, which is a reason why the loading efficiency of exosomes is comparatively lower [196]. Also, the use of methods like electroporation can lead to RNA aggravation, thereby reducing the loading efficiency of exosomes [201]. The lack of standardized methods for loading might also compromise the integrity, stability, and load capacities of these exosomes [206,207]. Culture conditions and the parent cell type are the other factors that influence the loading efficiency of exosomes [207]. Inefficient processing and storage of exosomes can lead to changes in size, composition, and physiology [193,194,200]. When stored under high temperatures, exosomes tend to show inconsistent patterns, while low temperatures result in cryodamage, affecting the therapeutic effects of the exosomes. Optimal storage conditions are required to maintain the size and stability of exosome composition [193,194]. Intracellular and microenvironmental factors like pH and acidity can influence the delivery capacity of exosomes [193].

Sterility is another challenge associated with exosome therapy. The similar size of viruses and exosomes makes it difficult to distinguish between them and contaminates the molecular composition of exosomes. For example, retroviruses such as human immunodeficiency virus-1 (HIV-1) and human T-lymphotropic virus type-1 (HTLV-1) use exosomes to spread throughout the body and compromise the immune system. The presence of these virus particles might lead to infections and cause changes in the parent cells [194]. Contamination from mycoplasma and other microbes can also alter the cellular properties of parent cells, giving inaccurate results [200]. The alterations in gene expression can also cause infections leading to biosafety issues [194,201]. The chances of developing thrombosis and hemostatic perturbations are also something that cannot be neglected. The presence of tissue factors and other procoagulants in exosomes derived from biofluids is one of the major factors that increase the risk of developing thrombosis [194]. The lack of specific methods to eliminate unwanted contents and determine the long-term safety of using exosomes is also a potential challenge [180].

## Conclusion and Future Perspectives

Exosomes is a tiny vesicles with diverse pathological and therapeutic functions. Understanding the exosome benefits will lead to preventing various neurological, immunological, and oncological diseases. Since, exosomes are very small and widely available in numerous sources of our biological system, choosing the appropriate sources is quite a complicated one. Because, their exosome research is an upcoming and emerging technique, the availability of literature is not sufficient to target the diseases. Also, the isolation technique needs proper standardization and it will vary depending on the sources of the exosomes, so understanding the isolation technique is also not fully developed.

Further, the uptake mechanism of exosomes varies depending on the sources, and biological and disease conditions. Proper uptake is ultimately required to bring the potential therapeutic efficacy to target the diseases. Since exosomes can be isolated from almost all the sources in our biological system, tumor cells also can produce a large number of exosomes, which promote and support tumor progression. In this way, exosomes derived from tumor cells are used as a biomarker to predict the severity of the tumor. Likewise, exosomes derived from stem cells, immune cells, and other biological fluids exert various therapeutic functions to treat cancer. Nowadays, bioengineered exosomes have also been introduced in the field of biomedical science for targeted therapy. Even though exosomes have multiple functions, there are certain hurdles and challenges present in the exosomes starting from the biogenesis, isolation, and uptake till achieve the physiological action. So, finding the solutions to overcome these challenges would be great to improve the therapeutic efficacy and suitability of exosomes. In the future, exosomes might be considered as one of the vital physiological agents to use as a potential delivery vehicle, and also to treat various diseases.

## Data Availability

All data generated or analyzed during this study are included in this published article.
